# Supplementary material for: Age-specific differences in the dynamics of protective immunity to influenza
Source: Nat Commun. 2019 Apr 10;10:1660. doi: 10.1038/s41467-019-09652-6 (PMC6458119; doi:10.1038/s41467-019-09652-6)
Supplement: Supplementary file 1 — Supplementary Information [file 41467_2019_9652_MOESM1_ESM.pdf]

Supplementary Information (SI)

Age-specific differences in the dynamics of protective immunity to influenza

Ranjeva *et al*.

Supplementary Tables

Subtype		Model	Log likelihood (SE)	ΔAICc
H1N1pdm09	Adults	with k	-79.5 (0.06)	0
		without k	-81.7 (0.03)	2.1
	Children	with k	-91.8 (0.01)	0
		without k	-95.9 (0.04)	5.6
H3N2	Adults	with k	-45.5 (0.04)	0
		without k	-48.6 (0.03)	3.6
	Children	with k	-77.8 (0.07)	0
		without k	-85.5 (0.05)	13.0

Supplementary Table 1: Model comparisons for sub-model of short-term boosting. Models with k include the antibody ceiling effect (Eq. 11, Supplementary Discussion, and models without k do not ($k_{a_i,s}$ = 0).

Subtype		Epidemic	Simulated incidence	From observed $\geq$ 4-fold changes
H1N1pdm09	Adults	1	0.09 [0.06, 0.16]	0.05
		2	0.12 [0.09, 0.15]	0.12
		3	0.07 [0.04, 0.09]	0.06
	Children	1	0.14 [0.09, 0.33]	0.10
		2	0.17 [0.12, 0.24]	0.19
		3	0.10 [0.05, 0.15]	0.06
H3N2	Adults	1	0.12 [0.09, 0.15]	0.16
		2	0.13 [0.11, 0.16]	0.15
		3	0.04 [0.03, 0.05]	0.03
	Children	1	0.22 [0.15, 0.30]	0.21
		2	0.23 [0.16, 0.30]	0.24
		3	0.06 [0.02, 0.13]	0.03

Supplementary Table 2: Incidence in each epidemic. Epidemics are shown in Fig. 3. The simulated incidence was estimated from the simulated latent infections. The main and bracketed values give the median and 95% quantiles, respectively, from 1000 replicate simulations of the models at the maximum likelihood estimate. Incidence was also estimated from the frequency of $\geq$ 4-fold titer consecutive titer rises observed in the data.

Infecting Subtype	Imprinting Group	Parameter	Population subset	MLE [95% CI]
H1N1pdm09	Group 1	($\alpha_{\mathrm{imp,H1N1pdm09}}$)	Ages 35 - 50 y	0.8 [0.6, 1.0]
H3N2	Group 2	($\alpha_{\mathrm{imp,H3N2}}$)	Ages 35 - 50 y	1.0 [0.9, 1.2]

Supplementary Table 3: Maximum likelihood estimates of the group-level imprinting effects ($\alpha_{\mathrm{imp,H1N1pdm09}}$ and $\alpha_{\mathrm{imp,H3N2}}$) among individuals ages 35-50 y. 95% confidence intervals (CIs) are reported.

	Notation	Type	Value
Parameter			
Magnitude of short-term boost	$d_{children,s}$<br>$d_{adults,s}$	Estimated (sub-model)	
Variability of short-term boost	$\sigma_{children,s}$<br>$\sigma_{adults,s}$	Estimated (sub-model)	
Antibody ceiling effect	$k_{children,s}$<br>$k_{adults,s}$	Estimated (sub-model)	
Magnitude of long-term boost	$\zeta_{children,s}$<br>$\zeta_{adults,s}$	Estimated	
50% protective titer (TP50)	$TP50_{children,s}$<br>$TP50_{adults,s}$	Estimated	
Weight of HI-correlated protection	$\psi_{children,s}$<br>$\psi_{adults,s}$	Estimated<br>Estimated	
Waning rate of non-HI-correlated immunity	$w_{nonspecific,children,s}$<br>$w_{nonspecific,adults,s}$	Estimated	
Waning rate heterosubtypic	$w_{nonspecific,m}$	Estimated	
Scaled transmission rate	$\beta_{scaled,s}$	Estimated	
Daily within-household transmission rate	ω_s	Estimated	
Age category-specific contact rate	β_{c,cat_i,cat_n}	Fixed	Contact matrix for five age categories [1]:<br>Young children (0-10)<br>Older children (11-20 y)<br>Young adults (21-40 y)<br>Older adults (41-65 y)<br>Elderly (>65 y)
Age distribution of community flu intensity	p_{cat_n}	Fixed	Young children: 0.19 [2]<br>Older children: 0.33<br>Young adults: 0.30<br>Older adults: 0.14<br>Elderly: 0.04
Infection duration	$\Gamma(\mu, \sigma_{dur})$	Fixed	μ = 5 d, σ_{dur} = 1 d [3]
Rate of short-term titer rise	r	Fixed	0.2 d^{-1} [4]
Duration: infection to peak titer	T_{peak}	Fixed	4 wks [4]
Rate of short-term titer waning	w	Fixed	0.008 d^{-1} [4, 5]
Scaling parameter of antibody protection curve	ϕ	Fixed	2.1 [6]
Measurement error	ϵ<br>$\epsilon_{undetectable}$	Fixed	1.29 [7, 8]<br>0.74 [7]
State variable			
Titer	$h_{i,s}(t)$	Simulated	
Baseline titer	$h_{baseline,i,s}(0)$	Fixed with error	
Susceptibility	$q_{i,s}(t)$	Simulated	
Time of infection	$t^X_{i,s}$	Simulated	

Supplementary Table 4: Model parameters and state variables.

Subtype		Parameter	MLE [95% CI]
H1N1pdm09	Adults	$d_{a_i,s}$	3.6 [2.4,5.2]
		$\sigma_{a_i,s}$	1.9 [1.4, 2.6]
		$k_{a_i,s}$	0.3 [0.1, 1.8]
	Children	$d_{a_i,s}$	3.5 [3.3, 5.5]
		$\sigma_{a_i,s}$	0.9 [0.7, 1.5]
		$k_{a_i,s}$	0.6 [0.3, 1.2]
H3N2	Adults	$d_{a_i,s}$	4.6 [3.1, 7.1]
		$\sigma_{a_i,s}$	1.4 [0.9, 2.3]
		$k_{a_i,s}$	1.0 [0.6 , 2.5]
	Children	$d_{a_i,s}$	5.1 [3.9,7.2]
		$\sigma_{a_i,s}$	1.5 [1.0, 2.2]
		$k_{a_i,s}$	0.5 [0.3, 1.0]

Supplementary Table 5: Maximum likelihood estimates of the parameters that govern the short-term titer dynamics with 95% confidence intervals (CIs).

Subtype		% Symptomatic infections	% Primary infections
H1N1pdm09	Adults	69.4%	38.9%
	Children	64.3%	54.8%
H3N2	Adults	76.2%	38.1%
	Children	69.0%	75.8%

Supplementary Table 6: Fraction of children and adults with symptomatic and primary infections for H1N1pdm09 and H3N2. Symptomatic infections are defined by an ARI in the two weeks before PCR-confirmed infection and primary infections are defined by the absence of infection with or without ARI symptoms in other household members in the two weeks before PCR-confirmed infection. ARI was defined as having least two of the following symptoms: fever $\geq$ 37.8°C, cough, sore throat, runny nose, headache, myalgia, and phlegm.

Subtype	Parameter	MLE [95% CI]
H1N1pdm09	$\beta_{\text{scaled,s}}$	-2.8 [-3.0, -2.7]
H3N2	$\beta_{\text{scaled,s}}$	-3.2 [-3.3, -3.1]

Supplementary Table 7: Maximum likelihood estimate of the subtype-specific scaled transmission rate, $\beta_{\text{scaled},s}$, for each subtype. Results are shown on a log scale with 95% confidence intervals (CIs).

Supplementary Figures

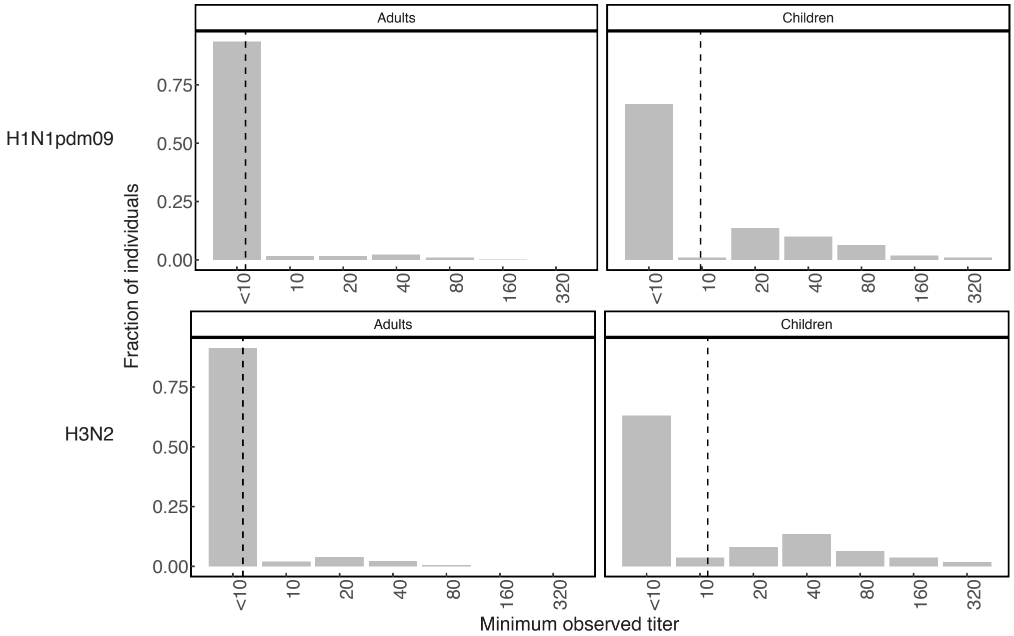

Supplementary Figure 1: Distribution of baseline titers. The distribution of the minimum observed (baseline) titers in children and adults for H1N1pdm09 and H3N2 are shown. The vertical dashed line gives the geometric mean baseline titer.

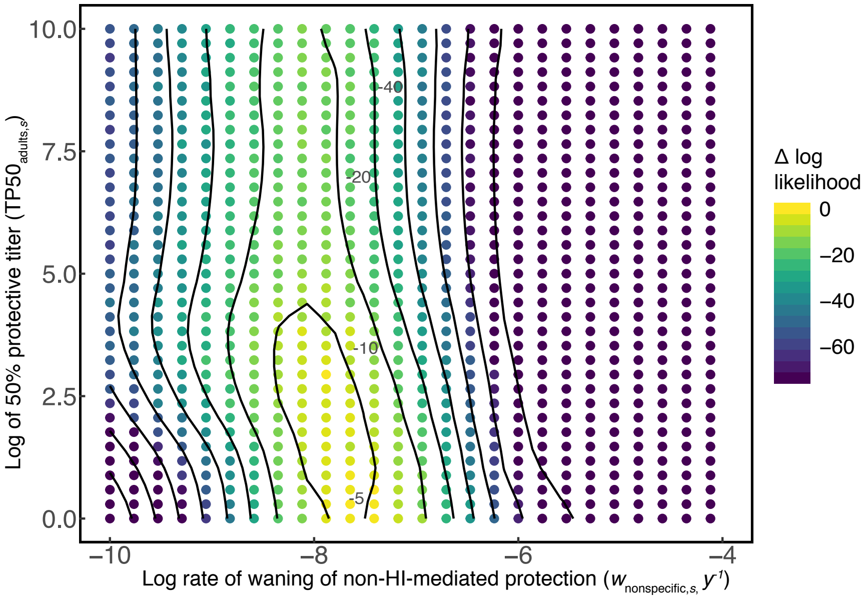

Supplementary Figure 2: Bivariate likelihood profile of the rate of waning of non-HI-mediated protection in adults and the 50% protective titer in adults ($TP50_{adults}$) for H1N1pdm09.

.

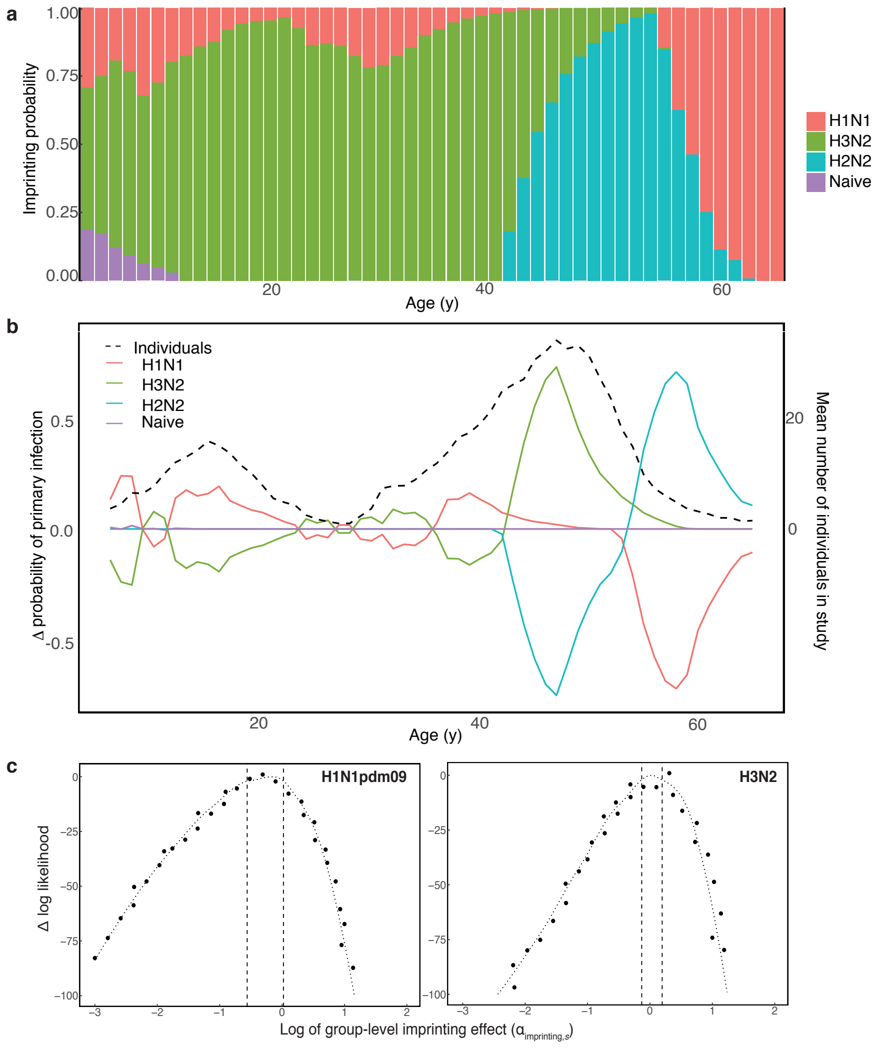

Supplementary Figure 3: Group-level imprinting. **(a)** Probability of imprinting by historically circulating influenza A subtypes by age in 2009. **(b)** Change in the mean probability of primary infection with historically-circulating subtypes by age between 2009 and 2014. The black dashed line gives the mean number of individuals by age that were observed in the data between 2009 and 2014. **(c)** Likelihood profiles for the effect of imprinting by H2N2 on the rate of infection with H1N1pdm09 (left) and the effect of imprinting by H3N2 on the rate of H3N2 infection (right). Values of the log parameter less than 0 (vertical dotted line) indicate a protective imprinting effect. The black dashed lines denote the 95% confidence interval.

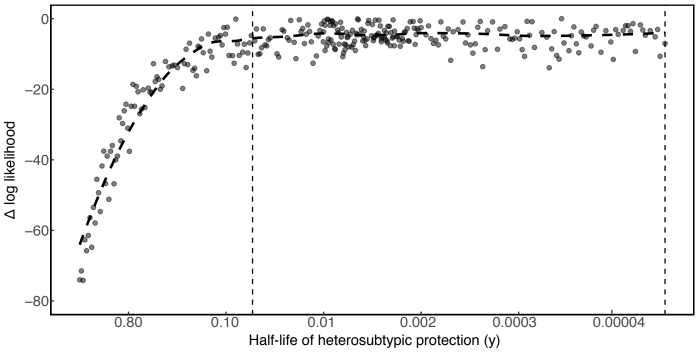

Supplementary Figure 4: Likelihood profile of the rate of waning of heterosubtypic protection. The x-axis gives the corresponding half-life of protection.

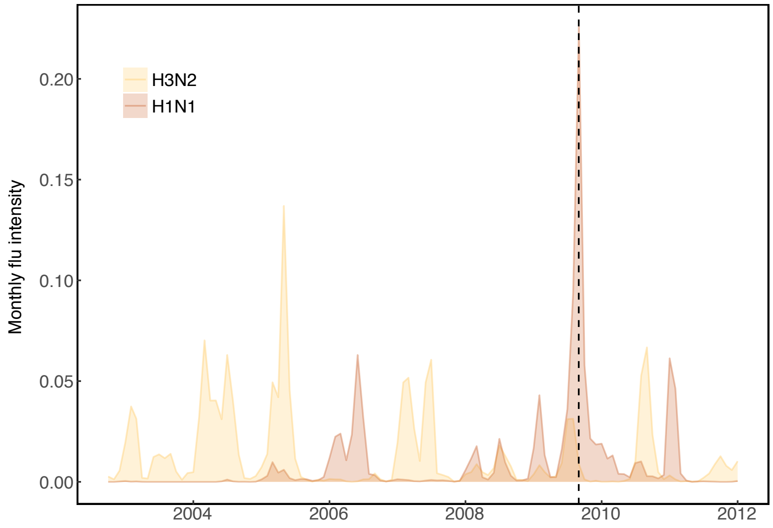

Supplementary Figure 5: Subtype-specific flu intensity (ILI $\times$ % positive) in Hong Kong. The black vertical dashed line denotes the earliest observation date in the data.

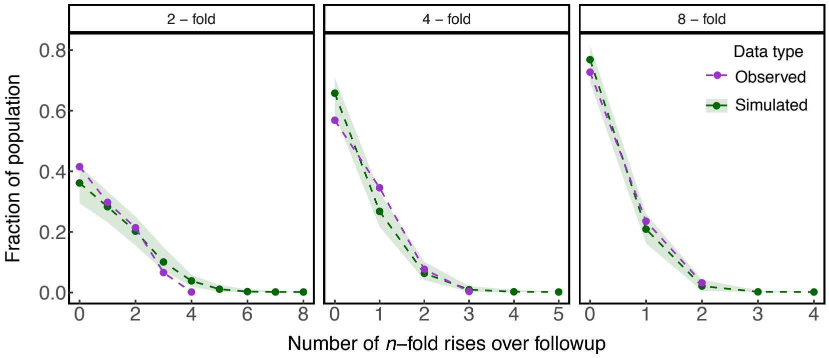

Supplementary Figure 6: Observed and simulated distributions of consecutive 2-, 4-, and 8-fold titer rises per individual in the H1N1pdm09 data. The dashed blue lines give the medians from 1000 replicate simulations of the model, and the shaded blue areas are bounded by the 2.5% and 97.5% quantiles.

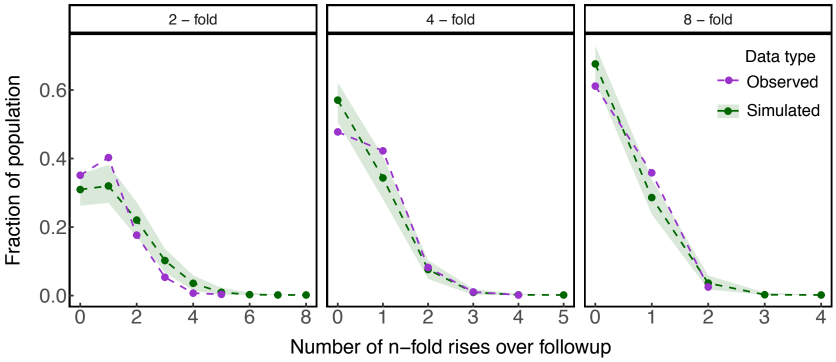

Supplementary Figure 7: Observed and simulated distributions of consecutive 2-, 4-, and 8-fold titer rises per individual in the H3N2 data. The dashed blue lines give the medians from 1000 replicate simulations of the model, and the shaded blue areas are bounded by the 2.5% and 97.5% quantiles.

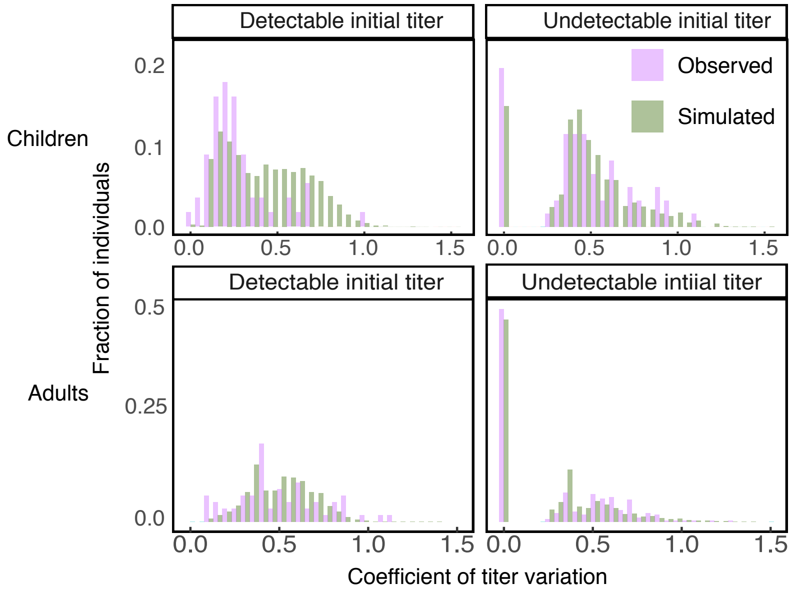

Supplementary Figure 8: Observed and simulated distributions of the coefficients of log titer variation in the H1N1pdm09 data. The distributions are shown for individuals with initial titers ≥ 10 (detectable) and for individuals with initial titers <10 (undetectable).

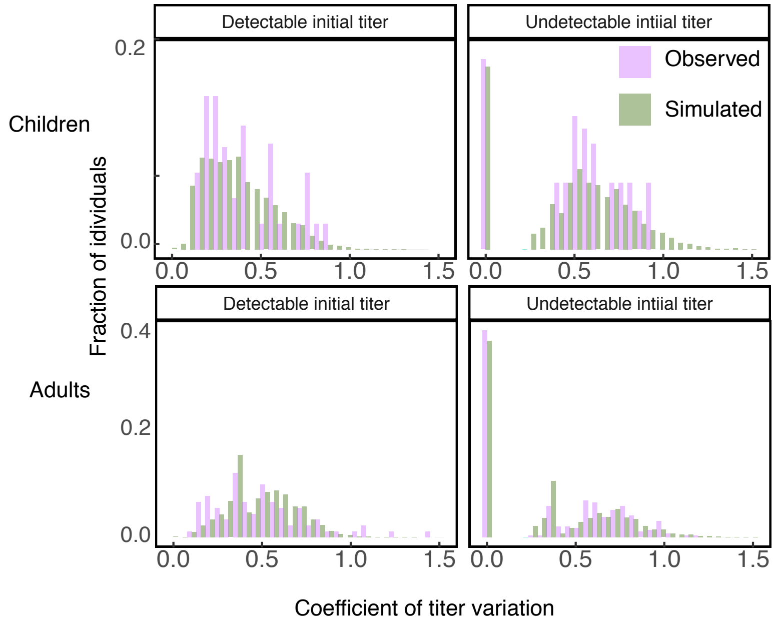

Supplementary Figure 9: Observed and simulated distributions of coefficients of log titer variation in the H3N2 data. The distributions are shown for individuals with initial titers ≥ 10 (detectable) and for individuals with initial titers <10 (undetectable).

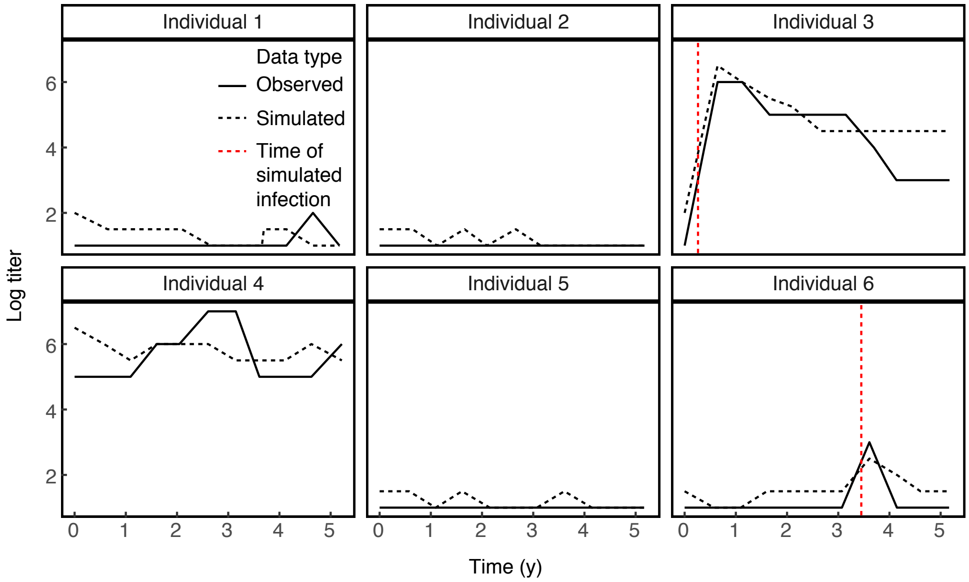

Supplementary Figure 10: Simulated individual trajectories from the filtered particle population of the model for H1N1pdm09 at the maximum likelihood parameters. The filtered trajectories were obtained using 50,000 particles. The solid and dashed black lines give the observed log titer and the filtered log titer trajectory, respectively. The dashed red lines denote latent times of infection from the model. Results are shown for the first six individuals in the dataset.

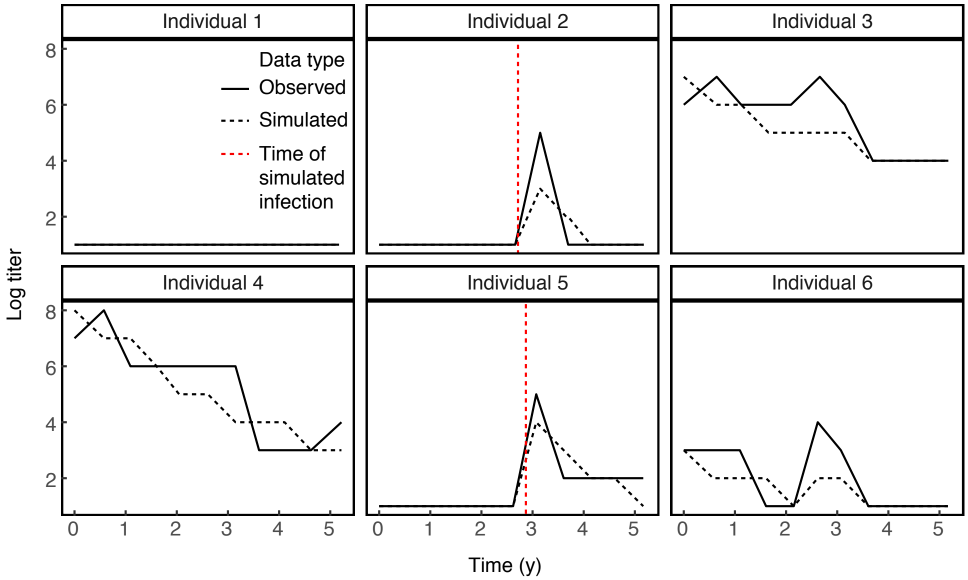

Supplementary Figure 11: Simulated individual trajectories from the filtered particle population of the model for H3N2 at the maximum likelihood parameters. The filtered trajectories were obtained using 50,000 particles. The solid and dashed black lines give the observed log titer and the filtered log titer trajectory, respectively. The dashed red lines denote latent times of infection from the model. Results are shown for the first six individuals in the dataset.

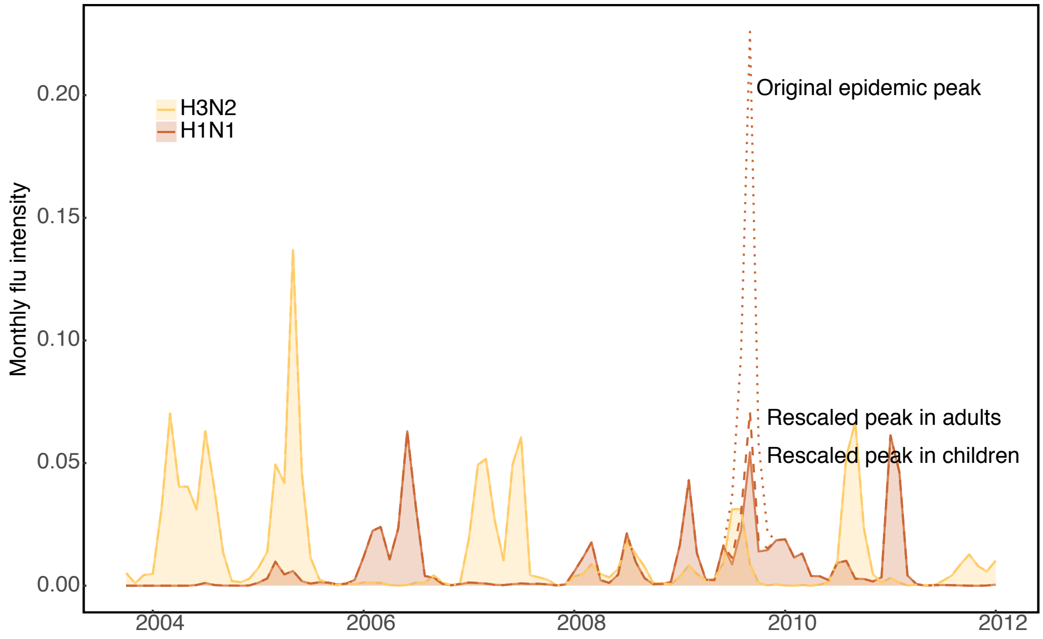

Supplementary Figure 12: Rescaled community intensity of H1N1pdm09 during the 2009 pandemic. Rescaled intensity shown in adults (dashed blue line) and in children (solid blue line and shading) compared to the original intensity reported by community surveillance (blue dotted line).

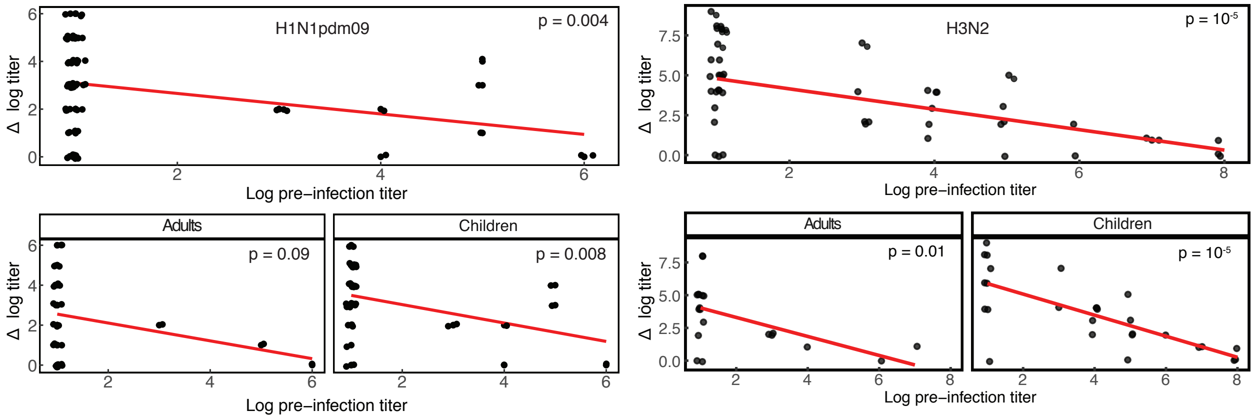

Supplementary Figure 13: Observed titer boost as a function of the pre-infection log titer for individuals with PCR-confirmed infection. Results are shown for H1N1pdm09 (left) and H3N2 (right). Boosts are calculated as the post-infection minus the pre-infection log titer. The top panel for each subtype gives the relationship for aggregated data from children and adults. Note that log titers are defined as in Eq. 18.

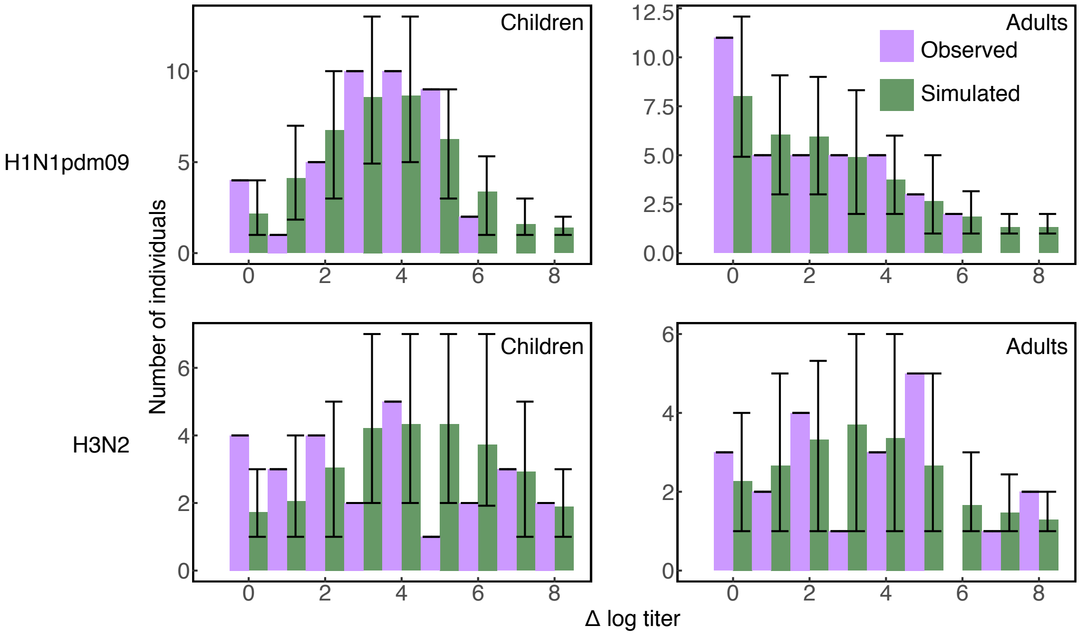

Supplementary Figure 14: Observed and simulated distributions of titer boosts from the sub-model in individuals with PCR-confirmed infections. Boosts are calculated as the post-infection minus the pre-infection log titer. Error bars give the 95% CI among simulations.

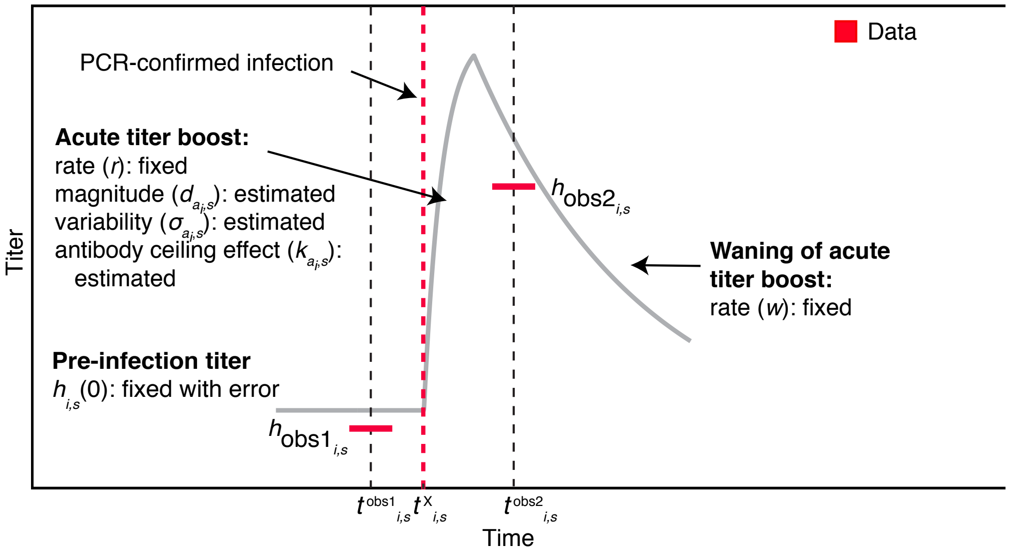

Supplementary Figure 15: Schematic of the acute HI titer dynamics for individual i against subtype s, given infection at time $t^{X}_{i,s}$. For the sub-model, $t^{X}_{i,s}$ is fixed based on the date of PCR-confirmed infection.

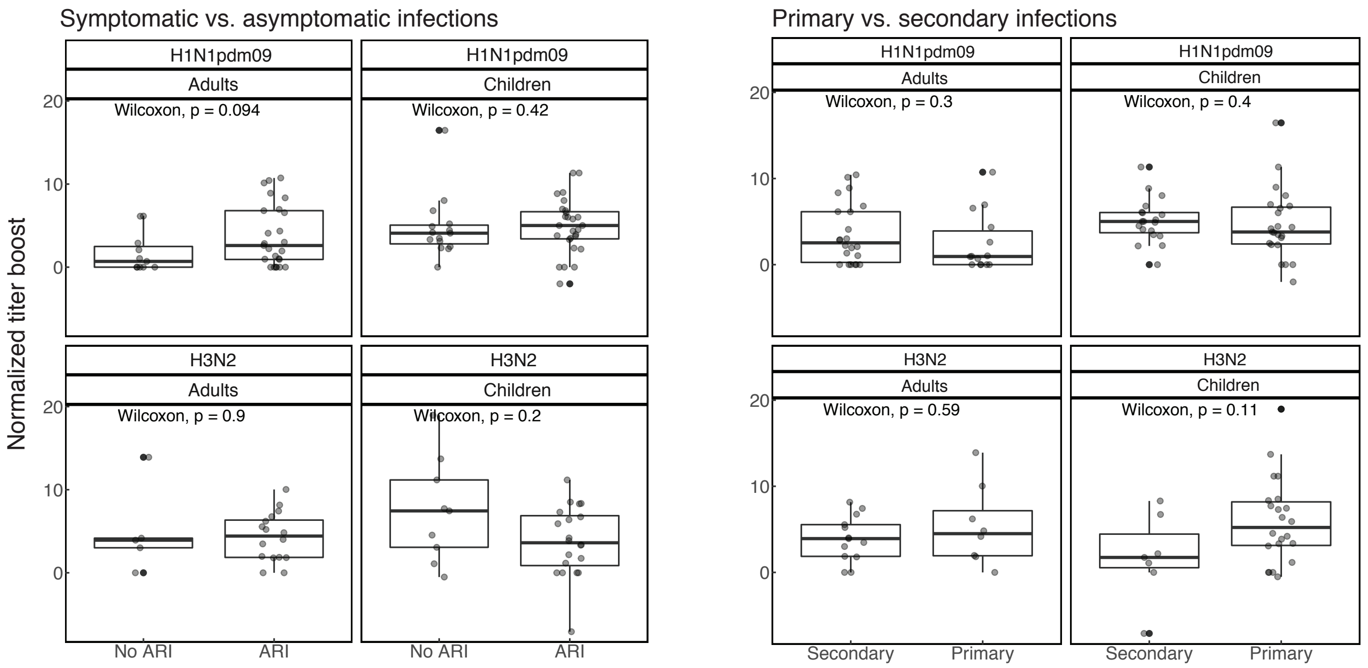

Supplementary Figure 16: Distribution of normalized titer boosts after PCR-confirmed infections. Results are shown for symptomatic and asymptomatic infections (left) and primary and secondary infections (right). Normalized titer boosts are calculated as the log post-infection titer minus the log pre-infection titer divided by the length of time in years between the pre- and post-infection samples. Box plots give the median and interquartile range of the normalized titer boosts, and the individual data points are overlain with horizontal jitter. Differences in the mean of the distributions are determined by non-parametric Wilcoxon tests.

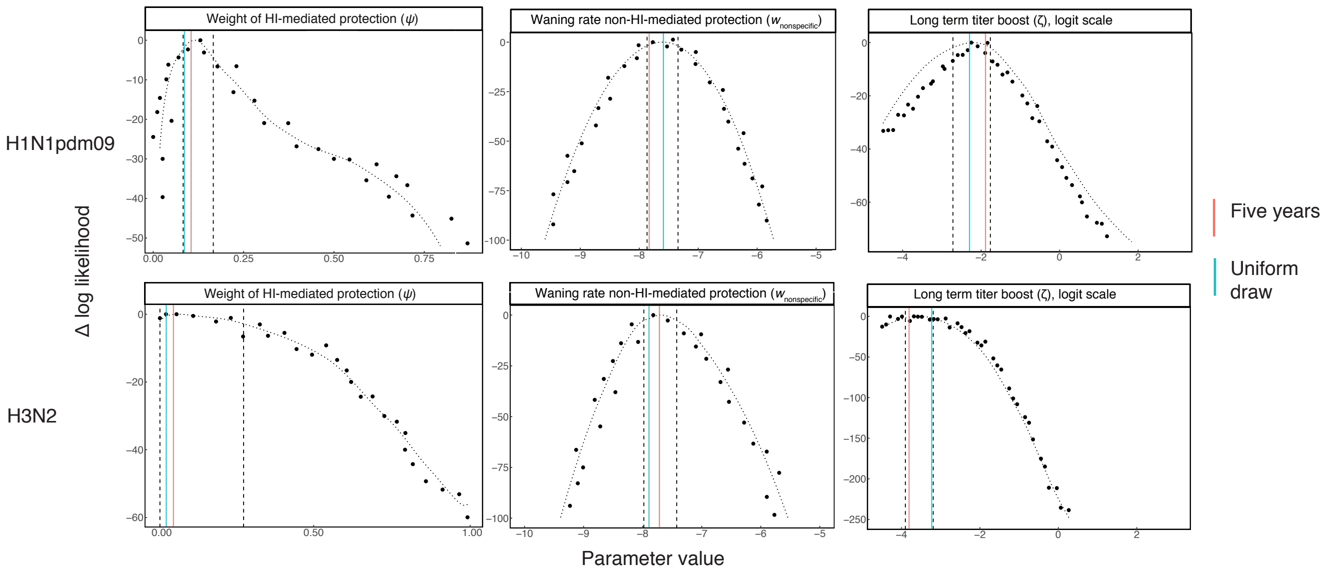

Supplementary Figure 17: Likelihood profiles for the estimated parameters of the best-fit models in **adults** for H1N1pdm09 and H3N2. The dashed curve gives the spline computed by the Monte Carlo Adjusted Profile technique (Supplementary Discussion). The vertical lines denote the MLEs from models under alternative initial conditions (Supplementary Discussion).

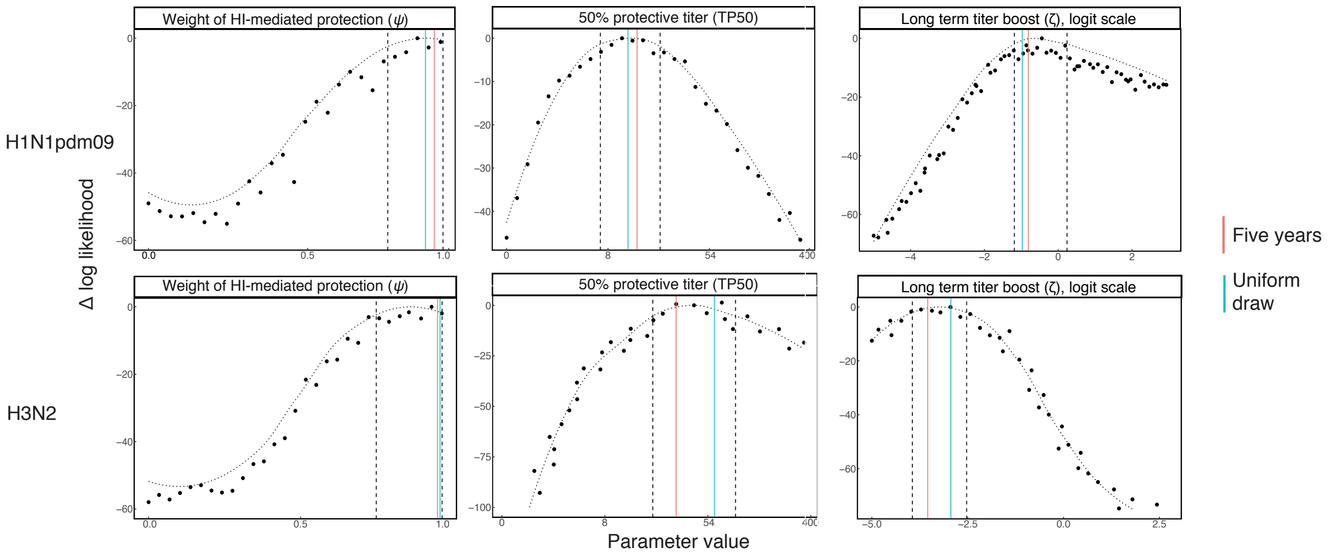

Supplementary Figure 18: Likelihood profiles for the estimated parameters of the best-fit models in **children** for H1N1pdm09 and H3N2. The dashed curve gives the spline computed by the Monte Carlo Adjusted Profile technique (Supplementary Discussion). The vertical lines denote the MLEs from models under alternative initial conditions (Supplementary Discussion) .

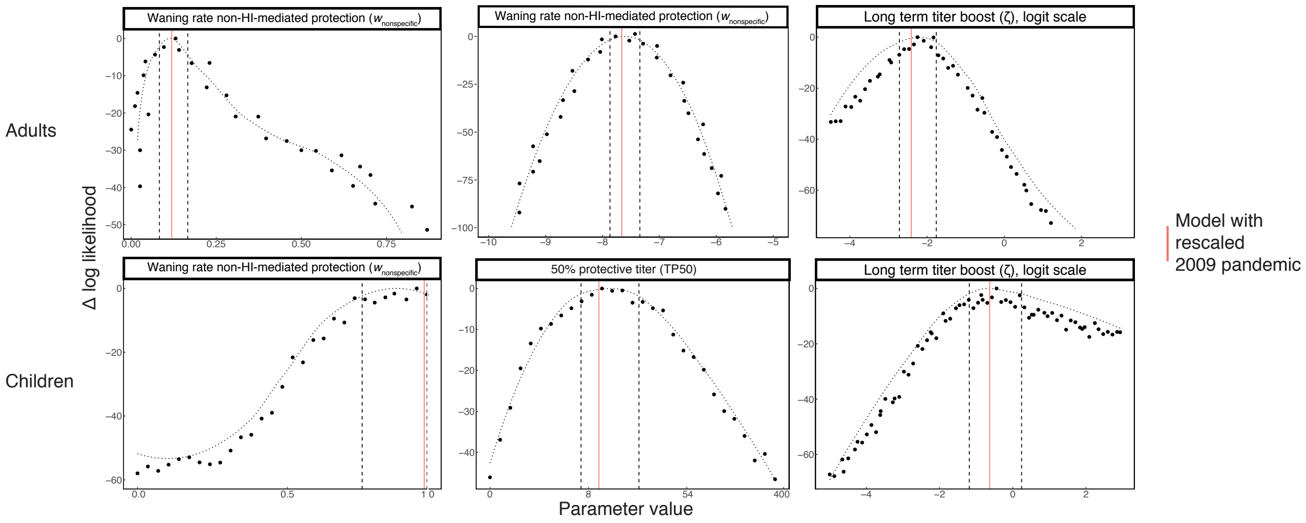

Supplementary Figure 19: Likelihood profiles for the estimated parameters of the best-fit models in adults and children for H1N1pdm09. The dashed curve gives the spline computed by the Monte Carlo Adjusted Profile technique (Supplementary Discussion). The vertical lines denote the MLEs from the model with rescaled H1N1pdm09 intensity during the 2009 pandemic (Supplementary Discussion) .

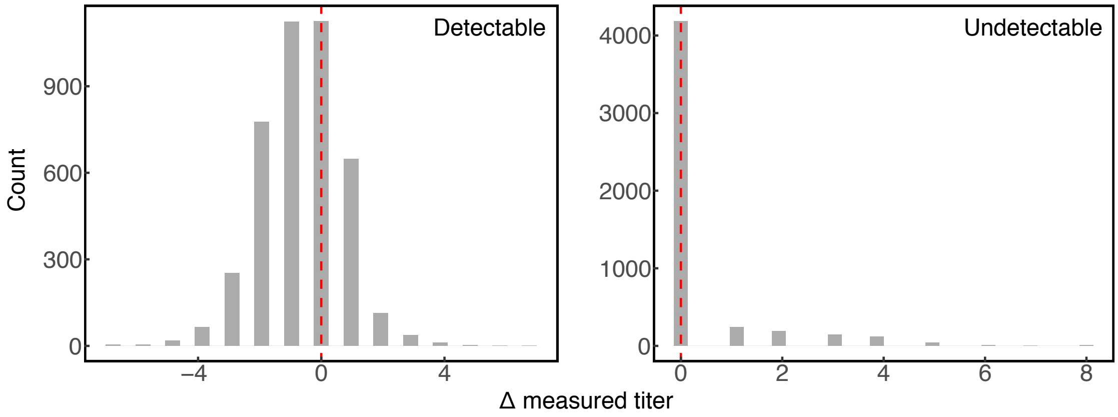

Supplementary Figure 20: Distribution of error from repeat sample measurements. Distribution of difference between the second and first measured titer for sera that were tested twice, with distributions shown separately for detectable and undetectable titers based on the initial measurement. The vertical red line marks zero difference.

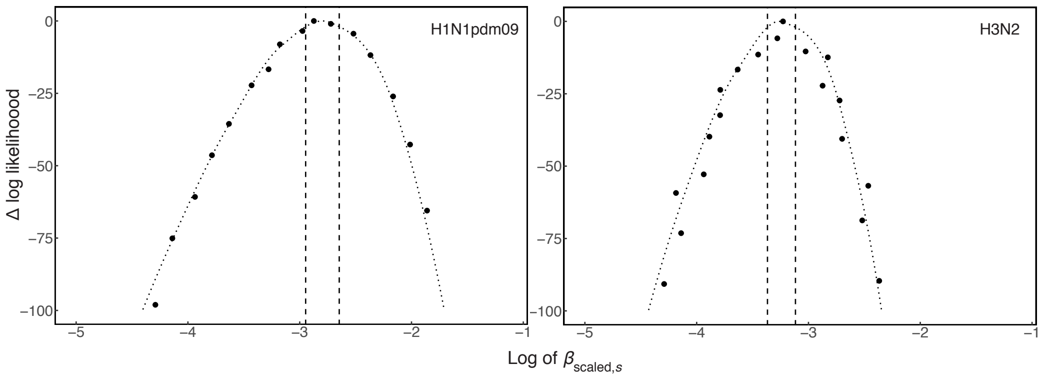

Supplementary Figure 21: Likelihood profiles for the subtype-specific scaled transmission rate, $\beta_{\text{scaled},s}$ for H1N1pdm09 and H3N2. The dashed horizontal line gives the threshold for statistical significance at a 95% level. The vertical lines denote the bounds of the 95% CI.

Supplementary Discussion

Short-term titer dynamics after PCR-confirmed infection

Model of short-term antibody boost after PCR-confirmed infection

To increase accuracy modeling the short-term post-infection titer dynamics (Eqs. 11 - 13, Fig. 1, Step 1a), we fit a sub-model to the observed titers before and after a PCR-positive swab (Supplementary Fig. 15). We estimate the mean magnitude and variability of the short-term titer boost ($d_{a_i,s}$ and $\sigma_{a_i,s}$, respectively) and the dependence of the boost on the pre-infection titer, $k_{a_i,s}$. This allows us to test for the presence of an antibody ceiling effect, which has been identified in studies of post-vaccination titer dynamics [9, 10].

To fit the sub-models, we fixed the pre-infection latent titer, $h_{i,s}(0)$, to the observed pre-swab titer, $h_{\text{obs1},i,s}$, allowing for two-fold uncertainty in the measured titer (Eq. 23). We fix the latent time of infection, $t^{\text{X}}_{i,s}$, based on the date of the positive swab, assuming that the swab occurred during an infected period that we draw from a gamma distribution with fixed parameters (Supplementary Table 4). We model the dynamics of the short-term titer rise as in Eq. 11, with the rate of rise r and time T_{peak} between infection and peak titer fixed (Supplementary Table 4). After peaking, the titer wanes at rate w (fixed as in Supplementary Table 4) until the time of the second observed value, $h_{\text{obs2},i,s}$.

Infection generates a variable short-term homosubtypic antibody boost that declines with increasing pre-infection titer.

The raw data suggest an antibody ceiling effect (Supplementary Fig. 13). We performed linear regressions of individuals' observed changes in log titer on their observed pre-infection log titers, excluding one individual with $\Delta t > 1$ y between the pre- and post-infection titer measurements. For both H1N1pdm09 and H3N2, the difference between pre- and post-infection log titers declines linearly with increasing pre-infection titer. The linear decline is statistically significant ($p<0.02$ for both subtypes). When we stratify the regressions in children and adults for each subtype, the decline is statistically significant, with $p \leq 0.01$ for children for both subtypes and for adults with H3N2 ($p=0.09$ for adults with H1N1pdm09).

The dynamical sub-models also support an antibody ceiling effect for both subtypes in children and adults (Supplementary Tables 1,5), such that higher pre-infection titer diminishes the boost. For both subtypes, models that include the antibody ceiling effect (with k) outperform models that do not (without k, $k_{a_i,s} = 0$) in children and adults (ΔAICc > 2, Supplementary Table 1). Therefore, part of the individual variation in the acute infection response can be explained by differences in pre-existing titers. Simulations from the MLEs of the best fit models of the short-term dynamics reproduce the shape of the observed distribution of titer boosts in children and adults after PCR-confirmed infection for both subtypes (Supplementary Fig. 14).

From the maximum likelihood parameter estimates of the best-fit sub-models, we find substantial variability in antibody titer responses after PCR-confirmed infection with both subtypes in children and adults (Supplementary Table 5). This finding is consistent with other analyses [11, 12]. The inferred standard deviation of the lognormal titer boost distribution (Eq. 12) ranges from 0.9 to 1.9 log titer units among children and adults for H1N1pdm09 and H3N2 (Supplementary Table 5). The mean magnitude of the boost is higher for H3N2 than for H1N1pdm09 in both age groups. The variability in the acute infection response and the difference in the response between subtypes and age groups suggest that threshold titers used in sero-surveillance may not reliably predict infection in all individuals [13, 6].

Observed titer boosts secondary to symptomatic vs. asymptomatic infections and primary vs. secondary infections

The sub-model of the short-term titer dynamics does not distinguish between symptomatic infections and asymptomatic infections that may have been detected incidentally given illness in another household member. If the measured titer boosts vary with symptom severity, our estimates may be biased, since index cases were identified by symptoms. We define symptomatic infection by the presence of ARI in the two weeks before PCR-confirmed infection. Based on the household symptom diaries, approximately 70% of infections in both children and adults for both subtypes were symptomatic (Supplementary Table 6). Children were more likely than adults for both subtypes to have a primary, or index case infection, meaning that no other household members had a PCR-confirmed infection or symptoms of an ARI in the two weeks prior to confirmed infection.

We compared the distributions of titer changes between symptomatic and asymptomatic infections and between primary and secondary infections (Supplementary Fig. 16). Because titers wane, we normalized the boost by the interval between the pre- and post-infection sample dates. We find no statistically significant difference in the mean normalized titer boost between symptomatic and aymptomatic infections for either subtype in children or adults. Similarly, we find no statistically significant differences when comparing primary and secondary infections. Therefore, the data suggest that the titer boosts estimated from PCR-confirmed infections in adults and children are not biased by differences in asymptomatic case detection.

Model validation and sensitivity analysis

The model reproduces the observed distribution of titer rises among individuals.

We compared the observed numbers of 2-,4-, and 8-fold increases in consecutive titer measurements for H3N2 and H1N1pdm09 to the distributions obtained from 1000 replicate simulations of the model at the MLEs (Supplementary Figs. 6, 7). The model reproduces the observed distributions in children and adults for both subtypes.

The model overestimates the variation in individuals' titers.

We compared the observed distribution of the coefficient of titer variation for individuals to the distribution obtained from 1000 replicate simulations of the model at the MLE (Supplementary Figs. 8, 9). We separately analyzed the distributions for individuals with detectable initial titers ($\geq$10) and undetectable initial titers (<10). In our models, any simulated titer <10 takes the value 10 of an undetectable titer. Therefore, the variation in undetectable titers by measurement error alone is less than that for titers $\geq$10. For both subtypes, the models tend to overestimate the individual variation over time. The bias is more pronounced among individuals with detectable baseline titers, which might be explained by the measurement error. Nevertheless, the difference in the means of

the observed and simulated CV distributions ranges from 0.0 to only 0.1 for children and adults with H1N1pdm09 and H3N2. Furthermore, the filtered particle population of the model, an estimate of the smoothed distribution of latent model variables, at the maximum likelihood parameters closely reproduces the observed titer trajectories for individuals (Supplementary Figs. 10, 11).

The maximum likelihood parameter estimates are robust to assumptions about the initial conditions.

To initialize the full model, we drew each individual's time of most recent infection from the density of the subtype-specific influenza intensity in the seven years preceding the first observation. For comparison, we fitted the best-fit model for each subtype in children and adults using two alternative assumptions about the initial conditions. First, we drew the time of most recent infection from the density of the subtype-specific influenza intensity over the five years before the first observation (Five years, Supplementary Figs. 17, 18). Second, we drew the time of most recent infection uniformly over the seven years before the first observation rather than using $L_s(t)$ (Uniform draw, Supplementary Figs. 17, 18). The maximum likelihood estimates of the alternative models fall within the 95% CI of the parameter estimates from the original assumption.

The inference results are robust to rescaling of the community intensity of H1N1pdm09 during the 2009 pandemic.

During the first wave of pandemic influenza H1N1pdm09 in 2009, increased reporting rates and changes in health-care seeking behaviors affected surveillance [14, 15]. We re-fitted our models of H1N1pdm09 after scaling the community flu intensity to adjust for these differences. A previous study estimated separate scaling factors for the relationship between the H1N1pdm09 intensity proxy and the rate of infection before and after a November 2009 change point [16]. We rescaled our estimate $L_s(t)$ of the 2009 pandemic H1N1pdm09 intensity by multiplying the intensity before the change point by the ratio ρ of the estimated post- and pre-change point scaling factors in children (ρ = 0.25) and adults (ρ = 0.29). Supplementary Fig. 12 shows the rescaled intensity. Notably, our observations begin at the end of the 2009 pandemic. Fewer than 6% of observations in children and fewer than 5% of observations in adults occurred before the November 2009 change point. Fewer than 1% of observations in children and adults occurred before October 2009. The model recovered the same MLE given the rescaled pandemic intensity (Supplementary Fig. 19).

The measurement error estimated from replicate titer measurements is consistent with literature estimates.

The sera from three visit dates were measured twice. In our models, we used the first titer measurement for each serum sample (the measurement recorded closest to the sampling date). To estimate the measurement error, we calculated the difference in measured titer between the second and first replicates (Supplementary Fig. 20). For detectable titer levels ($>$10), the standard deviation of the error distribution (SD = 1.23 log titer units) matches the measurement error that we fixed in the model according to estimates from the literature (Supplementary Table 4). The negative central tendency of the difference between the second and first replicates among detectable titers (median = -0.98 log titer units log titer units) indicates that measured titer generally declines with time since sampling. Additionally, in line with previous analyses [7], we find that the error distribution is zero-inflated for undetectable titers $<$10 (Supplementary Fig. 20), justifying our use of a separate measurement error for undetectable titers. A previous study estimated the probability of 2-fold measurement error for undetectable titers [7]. We therefore calculated the corresponding error (ϵ = 0.74) in our normally distributed observation model that would yield the same probability of 2-fold measurement error for undetectable titers. The observation model is non-invertible (Eq. 19). Therefore, while we use the measurement error to draw simulated observations from a normal distribution centered around the latent log titers, we cannot back-calculate the value of the latent titers from observed data. This is why we assign the initial baseline titer $h_{\text{baseline},i,s}(0)$ from a possible two-fold range surrounding the lowest observed titer $h^{\text{min}}_{\text{obs},i,s}$ (Eq. 23).

Age-specific contact rates

We used age-specific contact rates estimated from a population-based survey of social contact patterns in Hong Kong that recorded daily contacts from over 1100 individuals in five age categories (Supplementary Table 4, [1]). The authors calculated the relative number of contacts between individuals of each age category, adjusting for the propensity of individuals in each age class to respond to either paper or online questionnaires. The authors also reported the contact matrix between age groups. To fix the total daily number of contacts in our analysis for an individual of a particular age category, we multiplied the reported number of daily contacts from the reference group (children ages 0-10 years) by the relative number of contacts in each age category. To fix the number of daily contacts with individuals of each age group, we multiplied the total number of daily contacts by the fraction of age-group specific contacts from the contact matrix.

Historical influenza A subtype frequency data

Before 1968, annual subtype frequencies are specified by well-known durations of subtype circulation between historical pandemics [17]. After 1968, annual frequencies are calculated from subtype-specific surveillance data for Hong Kong or from Southeast Asia for years in which data from Hong Kong are unavailable. Between 1968 and 1997, subtype frequencies are the annual fraction of subtype-specific influenza A sequences in the Global Initiative on Sharing All Influenza Data (GISAID) database [18]. Aggregate regional data is used during years in which fewer than 30 sequences are available from Hong Kong or China. From 1997 to 2014, annual frequencies are the fraction of subtype-specific specimens reported by the Global Influenza Surveillance and Response System (GISRS) [19].

Monte Carlo error

A central feature of inference of stochastic models from large datasets is non-negligible Monte Carlo error that often makes it infeasible to calculate the likelihood with an error of less than one log likelihood unit. This principle holds especially for longitudinal (or panel) data, which often consist of a collection of individual time series that are dynamically independent apart from shared model parameters. Standard approaches for constructing 95% confidence intervals (CIs) rely on a threshold of 1.92 log likelihood units from the maximum log likelihood to construct the CI [20]. Therefore, high Monte Carlo error, or error in the likelihood calculation, also poses a challenge for estimating 95% confidence intervals. The Monte Carlo Adjusted Profile (MCAP) technique [21] shows that a quadratic approximation in the region of the maximum likelihood can be used to reliably extrapolate the 95% CI in systems with high Monte Carlo error. The MCAP algorithm accounts for the Monte Carlo error in the standard error of the spline fitted to a given likelihood profile. Importantly, Ionides and colleagues have shown that despite wider uncertainty around the maximum likelihood in systems with high Monte Carlo error, the MCAP approach reliably identifies the parameters. While the Monte Carlo variance of the log-likelihood estimate increases linearly with the amount of data, so too does the Fisher information, or the information about the system, and therefore the ability to reliably identify the maximum likelihood parameters.

In addition to using the MCAP technique to construct likelihood profiles, we accounted for Monte Carlo error in the maximum likelihood inference by initiating 100 independent MIF searches at random parameter values for any given model. To identify the MLE for a given point on a likelihood profile, we required that three MIF searches independently arrive within two log likelihood units of the maximum likelihood value.

Supplementary References

[1] Leung, K., Jit, M., Lau, E. H. Y. & Wu, J. T. Social contact patterns relevant to the spread of respiratory infectious diseases in Hong Kong. *Scientific Reports* **7**, 7974 (2017).

[2] Caini, S. *et al.* Distribution of influenza virus types by age using case-based global surveillance data from twenty-nine countries, 1999-2014. *BMC Infectious Diseases* **18**, 269 (2018).

[3] Fiore, A. E. *et al.* Prevention and control of influenza: recommendations of the Advisory Committee on Immunization Practices (ACIP), 2008. *MMWR. Recommendations and reports : Morbidity and mortality weekly report. Recommendations and reports* **57**, 1–60 (2008).

[4] Zhao, X., Ning, Y., Chen, M. & Cook, A. R. Individual and population trajectories of influenza antibody titers over multiple seasons in tropical Singapore. *American Journal of Epidemiology* (2017).

[5] Yuan, H.-Y. *et al.* The impact of stratified immunity on the transmission dynamics of influenza. *Epidemics* **20**, 84–93 (2017).

[6] Coudeville, L. *et al.* Relationship between haemagglutination- inhibiting antibody titres and clinical protection against influenza: development and application of a bayesian random-effects model. *BMC Medical Research Methodology* **10**, 18 (2010).

[7] Cauchemez, S. *et al.* Influenza Infection Rates, Measurement Errors and the Interpretation of Paired Serology. *PLoS Pathogens* **8**, e1003061 (2012).

[8] Kucharski, A. J., Lessler, J., Cummings, D. A. & Riley, S. Timescales of influenza a/h3n2 antibody dynamics. *PLoS biology* **16**, e2004974 (2018).

[9] Jacobson, R. M. *et al.* Profiles of influenza A/H1N1 vaccine response using hemagglutination-inhibition titers Robert. *Human Vaccines & Immunotherapeutics* **11**, 961–969 (2015).

[10] Ohmit, S., Petrie, J. & Cross, R. Influenza hemagglutination-inhibition antibody titer as a correlate of vaccine-induced protection. *The Journal of Infectious Diseases* **204**, 1879–1885 (2011).

[11] Freeman, G. *et al.* Quantifying homologous and heterologous antibody titre rises after influenza virus infection. *Epidemiology and infection* **144**, 2306–16 (2016).

[12] Fonville, J. M. *et al.* Antigenic Maps of Influenza A(H3N2) Produced With Human Antisera Obtained After Primary Infection. *Journal of Infectious Diseases* **213**, 31–38 (2016).

[13] Hobson, D., Curry, R. L., Beare, A. S. & Ward-Gardner, A. The role of serum haemagglutination-inhibiting antibody in protection against challenge infection with influenza A2 and B viruses. *The Journal of Hygiene (London)* **70**, 767–77 (1972).

[14] Cowling, B. J. *et al.* Incidence of Influenza Virus Infections in Children in Hong Kong in a 3-Year Randomized Placebo-Controlled Vaccine Study, 2009-2012. *Clinical Infectious Diseases* **59**, 517–524 (2014).

[15] Marmara, V., Cook, A. & Kleczkowski, A. Estimation of force of infection based on different epidemiological proxies: 2009/2010 Influenza epidemic in Malta. *Epidemics* **9**, 52–61 (2014).

[16] Tsang, T. K. *et al.* Association Between Antibody Titers and Protection Against Influenza Virus Infection Within Households. *The Journal of Infectious Diseases* **210**, 684–692 (2014).

[17] Kilbourne, E. D. Influenza pandemics of the 20th century. *Emerging infectious diseases* **12**, 9–14 (2006).

[18] Shu, Y. & McCauley, J. GISAID: Global initiative on sharing all influenza data - from vision to reality. *Eurosurveillance* **22**, 30494 (2017).

[19] Flahault, A. *et al.* FluNet as a Tool for Global Monitoring of Influenza on the Web. *JAMA* **280**, 1330 (1998).

[20] Bolker, B. Likelihood and all that. In *Ecological Models and Data in R*, chap. 6, 169–221 (Princeton University Press, 2007), 508 edn.

[21] Ionides, E. L., Breto, C., Park, J., Smith, R. A. & King, A. A. Monte Carlo profile confidence intervals for dynamic systems. *Journal of The Royal Society Interface* **14**, 20170126 (2017).
